# Marfan syndrome in adolescence: adolescents’ perspectives on (physical) functioning, disability, contextual factors and support needs

**DOI:** 10.1007/s00431-019-03469-7

**Published:** 2019-10-16

**Authors:** Jessica Warnink-Kavelaars, Anita Beelen, Tine M. H. J. Goedhart, Lisanne E. de Koning, Frans Nollet, Mattijs W. Alsem, Leonie A. Menke, Raoul H. H. Engelbert

**Affiliations:** 1 Amsterdam UMC, University of Amsterdam, Rehabilitation, Amsterdam Movement Sciences, Meibergdreef 9, PO 22660, 1100 DD Amsterdam, Netherlands; 2 Department of Rehabilitation, Physical Therapy Science & Sports, UMC Utrecht Brain Center, University Medical Center Utrecht, Utrecht, the Netherlands; 3 Center of Excellence for Rehabilitation Medicine, UMC Utrecht Brain Center, University Medical Center Utrecht, and De Hoogstraat Rehabilitation, Utrecht, the Netherlands; 4 ACHIEVE, Center of Applied Research, Amsterdam University of Applied Sciences, Faculty of Health, Amsterdam, the Netherlands; 5 Amsterdam UMC, University of Amsterdam, Pediatrics, Meibergdreef 9, Amsterdam, Netherlands

**Keywords:** Marfan syndrome, Adolescence, Qualitative research, Participation, Connective tissue diseases, International classification of functioning, Disability and health for children and youth

## Abstract

Although essential for providing optimal adolescent patient support, knowledge of the impact of Marfan syndrome in adolescence is limited. To explore adolescents’ perceived impact of Marfan syndrome on (physical) functioning (activities, participation), disability (limitations, restrictions), contextual factors and support needs, we interviewed 19 adolescents with Marfan syndrome. Audio-recordings were transcribed, coded and analysed using thematic analysis. Identified themes were “difficulties in keeping up with peers” and “being and feeling different from peers”. Furthermore, an adolescent Marfan syndrome-specific International Classification of Functioning, Disability and Health for Children and Youth (ICF-CY) model derived from the data describing the adolescent perceived impact of Marfan syndrome on functioning, disability and its contextual factors. Adolescents perceived problems in keeping up with peers in school, sports, leisure and friendships/relationships, and they could not meet work requirements. Moreover, participants perceived to differ from peers due to their appearance and disability. Contextual factors: coping with Marfan syndrome, self-esteem/image, knowledge about Marfan syndrome, support from family/friends/teachers, ability to express needs and peer-group acceptation acted individually as barrier or facilitator for identified themes.

*Conclusion*: Adolescents with Marfan syndrome perceived limitations and restrictions in (physical) functioning. They perceived problems in keeping up with peers and perceived to differ from peers due to their appearance and disability. This warrants awareness and tailored physical, psychosocial, educational and environmental support programmes to improve (physical) functioning and empowerment of adolescents with Marfan syndrome.

**Table:** 

**What is known:** *• Marfan syndrome is a hereditary connective tissue disorder.* *• Marfan syndrome affects multiple systems.*	
**What is new:** *• Adolescents with Marfan syndrome perceive (1) problems in keeping up with peers in school, sports, leisure, friendships/relationships and work (2) to differ from peers due to their appearance and disability.* *• An adolescent Marfan syndrome-specific International Classification of Functioning, Disability and Health for Children and Youth model derived from the data describing the adolescent perceived impact of Marfan syndrome on functioning, disability and contextual factors.*

**Electronic supplementary material:**

The online version of this article (10.1007/s00431-019-03469-7) contains supplementary material, which is available to authorized users.

## Introduction

Marfan syndrome (MFS) is a rare hereditary connective tissue disorder caused by a mutation in the *FBN1* gene. The incidence is approximately 2–3 in 10,000 worldwide [18]. Manifestations of MFS in body structures and functions are well described, and the MFS diagnosis relies on defined clinical criteria (revised Ghent nosology) [18]. A markedly variable phenotype can be present in MFS, and a different number of disabilities can appear, even in the same family [2, 8, 18, 28, 29, 36]. Although essential for providing optimal adolescent patient support, studies on the overall impact of MFS on (physical) functioning (activities, participation), disability (limitations, restriction) and contextual factors during adolescence are sparse. Current studies reported significant burden on physical activities [7, 27, 28, 30], schooling and job opportunities [33], low work participation [34] and self-image [27] in adolescents and young adults with MFS. Then, a review showed that MFS had a significant impact on psychosocial aspects: decreased quality of life, education, work and family life, depression and anxiety [35]. Moreover, our recent qualitative study in parents on the perceived impact of MFS on daily functioning of their children aged 4–12 years with MFS showed that their children with MFS could not keep up with peers and experienced participation restrictions and unsupportive attitudes towards their physical appearance related to MFS [37].

Furthermore, studies on (physical) functioning of children and adolescents with related congenital heart and connective tissue diseases showed the limiting impact of disease-related physical problems on physical activities [1, 9, 32] and the negative impact on school attendance and extracurricular activities [9], play and leisure [4] and school participation [15]. Moreover, MFS and cardiovascular diseases guidelines gave some advice on physical activity and sport participation [19].

Current literature showed limited knowledge of the impact of (MFS) in adolescence. The first aim of this qualitative study is to explore adolescents’ perceived impact of MFS on (physical) functioning (activities, participation), disability (limitations, restrictions), contextual factors and support needs. The second aim is to develop an adolescent MFS International Classification of Functioning, Disability and Health for Children and Youth (ICF-CY) model describing the impact of MFS on adolescents (physical) functioning and disability. The indicated themes and the adolescent MFS-specific ICF-CY model may improve communication and awareness of the adolescent’s perceived impact of MFS by medical staff and related health professionals, the adolescents with MFS themselves and their relatives and friends which could prevent under-recognition and under-treatment. This requires individually tailored physical, psychosocial, educational and environmental support programmes to improve (physical) functioning and empowerment of adolescents with MFS.

## Materials and methods

### Participants and sampling strategy

The Medical Ethics Review Committee of the Amsterdam University Medical Centers, in the Netherlands has waived ethical approval under Dutch Law (reference number W17_054#17.071). Adolescents with MFS aged 12–18 years, treated at the Amsterdam University Medical Centers, were recruited by letter and selected [12] for diversity of age and gender. Participation in this study was voluntary and written informed consent was obtained from all participants.

### Interviews

The main interview framework was a newly developed question guide for semi-structured interviews (Table 1) based on topics collected from relevant studies on MFS and other connective tissue and related disorders, clinical experiences and remarks from Dutch and European MFS patient associations. This list was checked by a psychologist (qualitative research expert); no alterations were made. Data saturation was expected after a sample size of 14–18 interviews [12]. This point in data collection, when no new additional data for new themes were found, was based on the literature, the complexity of the research question and the diversity of the sample. Field notes were made. Adolescents could choose an interview with a paediatric rehabilitation physician (JW-K, MD, female) alone or in the presence of their parents. Parents were asked not to interfere.

**Table 1:** Question guide for semi-structured interviews

Question guide for semi-structured interviews	
What do you know about MFS?	
How did you gather this information?	
Which features of MFS do you have yourself?	
What is the impact of MFS on your activities? What is the impact of MFS on your participation in daily life?	
What are your concerns about your daily life (related to MFS)?	
How do you manage and cope with your limitations, restrictions and concerns (related to MFS)? What kind of physical or emotional support do you get and what helps you to participate in daily life?	
Who and how did you tell about MFS?	
What is the attitude of peers and other people towards your disease?	
What is the impact of MFS on your family life?	
Have you thoughts about your future adult life (work, relationships, family life, health, leisure)?	
Which supplementary (medical) support do you need and what is your advice to optimise adolescent MFS care?	

### Data analysis

Data were analysed with a thematic analysis approach [3]. Audio-recordings were transcribed, and concepts were coded by two investigators (JW-K, MD, female; TG, MD, female) using qualitative analysis software (MAXQDA 12 sfqda, 1989–2018, VERBI Software–Consult–Sozialforschung GmbH, Berlin, Germany). During the process of data collection, identified codes were validated in successive interviews until saturation was reached [12]. The calculated intercoder agreements served primarily to improve codes and coding instructions. Codes were structured to the domains of the ICF-CY [6], which offers a conceptual model for recording problems manifested in childhood and adolescence involving body functions and structures, activity limitations, participation restrictions and contextual factors. Themes were identified as well as contextual factors acting as a barrier or facilitator for identification. The adolescents’ reported support needs were categorised.

### Strategies to ensure trustworthiness and credibility

Three investigators (JW-K, MD, female; TG, MD female; AB, PhD, female) ensured trustworthiness and credibility throughout the data collection and analysis process [21]. Investigator triangulation was used to ensure rigour [21]. The manuscript reporting adheres to consolidated criteria for reporting qualitative research (COREQ), guidelines for reporting qualitative studies [see additional file 1] and the Standards for Reporting Qualitative Research [23, 31].

## Results

### Participant characteristics

Of the 21 adolescents with MFS approached to participate in the study, 2 declined, 1 due to lack of time and 1 gave no specific reason. Nineteen adolescents gave written informed consent and were interviewed between March 2017 and March 2018. In all participants, a pathogenic *FBN1* variant was confirmed. Fifteen participants had a parent diagnosed with MFS (see Table 2).

**Table 2:** Participants’ characteristics

Total participants	19
Gender (male/female)	12/7
Age (range) (years)	14.5 (12–17)
Confirmed pathogenic *FBN1* variant	19
Participant with parent with MFS	15
Ectopia lentis	9
*Z* score > 3 aortic root dilatation	4
Mitral valve prolaps	9
Heart medication	11
Systemic Ghent score ≥ 7	10
Beighton ≥ 6	10
Aorta operation	1
Lens operation	2
Foot operation	1
Secondary education level: (low vocational/middle vocational/higher general/pre-university education)	0/5/6/8

No missing data

### Interviews

All 19 interviews were conducted at Amsterdam University Medical Centers; 7 with the adolescent alone, 10 in the presence of one parent, and 2 in the presence of both parents. The interviews lasted between 30 and 75 min. Data saturation was reached after 14 interviews; no additional codes were identified in 5 successive interviews. After that, enrolment stopped. The intercoder agreement for the interviews was high for code existence and code frequency [mean (range) 85.9%, (77.5–91.1%) and 81.0% (71.3–87.8%), respectively].

### Themes

Identified themes related to (physical) functioning and disability were “difficulties in keeping up with peers” and “being and feeling different from peers” (see Table 3).

**Table 3:** Overview of themes on the impact of MFS on functioning, disability and contextual factors acting as a barrier or facilitator supported by the data: quotes from adolescents with MFS aged 12–18 years

Themes		Quotes
Difficulties in keeping up with peers	School	“Yes, Marfan’s does take up a part of my life, because I have to go to the hospital very often, for appointments and other things. I try to schedule my visits to the hospital so they do not conflict with school, and I do not have to miss any classes, but that is difficult. It is usually very busy at the hospital, and the appointments have to take place during school hours. This means I miss many classes. And I cannot participate in a lot of activities during Phys-ed.” A6
	Sports	“I really notice that I am not always able to participate. In sports, in particular, I often have to give up earlier. My knees ache a lot, so many times I stop before we are done.” A8
	Leisure	“I had problems last year; we went to Disneyland in Paris, and I was exhausted after only an hour and a half. At the time, my back, ankles and knees were really bothering me.” A13
	Friendships/relationships	“Well most of the time I am too tired to meet with friends after school and then, at home, I sit down on the couch to relax. So I do not really have time to meet with someone, that’s hard, but I usually play a game on my phone or I play online games together with friends. “A8
	Work	“At first, I had a part-time job at the local drugstore, stocking shelves, but I had to quit because my back was causing a lot of pain. So, I had to make a decision. I mean, it is a shame, because it was nice to earn some money, but my health is my main priority.” A5
Being and feeling different from peers	Appearance	“I am taller and thinner than the other kids in my class, and I have spider hands.” A10
	Fatigue	“I went to a concert with some of my friends, and I was really very tired. I was thinking “this is not right,” but on the other hand, I realized that I had had a really good day, so...” A2
	Pain	“Yes, the pain makes me unable to bend my hand fully. I can now, but I was not able to do it yesterday, and usually, it stays that way for a couple of days. I do not know if I strained it, or if it is caused by too little connective tissue, but when it happens, I really have a lot of pain in my wrist. I often drop my phone, I just lose my grip on it.” A1
	Activities	“I have an elevator pass that allows me to use the elevator, because taking the stairs is too hard, and I have a second set of books, so I do not have to carry them from home to school. The second set is kept at school, in my locker, so my bag is lighter. It prevents me from having to lift things, and keeps me from getting tired so quickly.” A3 “I am able to clean my room or do things like that, but when we go grocery shopping, my mother will tell me I do not have to carry the heavy items because I cannot.” A12
	Feeling different	“It bothers me sometimes, being an exception. I am insecure because you can see that we are very tall or very thin, and people will notice the dent in my chest, and ask me “what’s wrong with you?”; It’s not always nice, having to tell them you have Marfan’s, and I have to keep telling them over and over.” A4
Contextual factors reportedly acting as a barrier or facilitator		
Coping with MFS	Acceptation	“In the beginning, when I was younger, I had a hard time dealing with the fact that I have Marfan’s. But then I accepted the fact that I have an illness, and this is here to stay for the rest of my life, it is not going to change or anything. So accepting it really helped, and nowadays it’s no longer a problem for me.” A6
	Humorous and relaxed outlook on life	“Yes, I was disappointed when I had to give up basketball (highest junior league) because of Marfan’s because I really liked playing. But it’s in my character to try and make the best of the situation. So yes, now I am going to try to become as good as I can in my music.” A10
	Pro-active/planning	“I plan a lot in advance. For instance, if I know there are four tests (at school), I start preparing early, so I have less to do each day, and not two whole paragraphs on one day. That is too exhausting, and I get a headache if I do too much.” A18
	Avoidance and denial	“I really do not want to have anything to do with Marfan’s. I quickly go to the hospital, and that’s it for me.” A7
Self-esteem/image		“When I went swimming, people would react to my chest, saying things like “look at that” and other things. Yes, it bothered me a lot, and it prevented me from going sometimes. I would think “I really cannot deal with this now” and not go.” A5
MFS knowledge		I know you get thinner when you have Marfan’s, you can break bones easier, and your eyes can be more sensitive. And yes, your aorta also grows, or something. Other than that, I do not really know all that much about it.” A19
Ability to express needs		“If something happens to me, I think I would like to be able to talk to someone who also has Marfan’s and who has experienced the same thing.” A15
Support and peer group acceptance	Friends	“Yes, I have explained what Marfan’s is to my friends, so they understand it completely, and they are considerate. One of them actually just sent me a message, wishing me good luck today.” A14
	Parents	“Yes, when I am worried, I can talk to my sister, and to my parents.” A18
	Teachers/school	“I was unable to take the stairs or to keep up with the rest during the Phys-ed classes. I also had trouble studying, because there were too many stimuli at school, and after school, I was so tired, so basically, I just slept a lot. And I always had too little time to study or to do my homework. Now, at my new school, they understand that I have Marfan’s, and I get a lot of guidance and support. I am doing a lot better now.” A13

*MFS*, Marfan syndrome

### “Difficulties in keeping up with peers”

“I really notice that I am not always able to participate. In sports, in particular, I often have to give up earlier. My knees ache a lot, so many times I stop before we’re done.” *A8*

Adolescents perceived problems in keeping up and participating with peers in school, sports, leisure, friendships/relationships, and they could not meet work requirements. Time-consuming medical visits and treatments made it more difficult to participate in activities with peers.

#### School

Adolescents perceived difficulties in continuing a full school day, completing their school assignments and participating in gymnastics and leisure. This limiting impact of pain, fatigue, medical visits and treatment appointments on their pace of school work was hardly taken into account by their teachers.

#### Sports

Adolescents reported they could not keep up with their peers during sports due to physical impairments and limitations in physical activities. Fear of increased musculoskeletal injuries, pain and fatigue and aorta and eye problems were mentioned. Alternatively, adolescents focused on individualised sports or implemented a physical activity in their schedule.

#### Leisure

Leisure activities such as (visits to) parties, theme parks, concerts and holidays were perceived as too exhausting; they often cancelled. Adolescents tried to find achievable leisure activities to participate in with peers.

#### Friendships/relationships

Maintaining friendships was perceived as complicated. Accompanying friends to (physical) activities, such as meeting at sport clubs, hiking or shopping was not always feasible due to fatigue and pain. Adolescents also addressed difficulties in making new friends, and reported feeling insecure about themselves. Out of 19 adolescents with MFS, two were into a relationship, both with a healthy partner. Adolescents responded that they had not met the right person yet or had no time for dating.

#### Work

Adolescents reported they could not meet the same work requirements as their peers. Working in a supermarket, restaurant, bar or shop was physically demanding because of regular carrying and/or lifting. Long hours standing and walking were considered challenging because of increased pain and fatigue. Adolescents selected physically less-demanding jobs such as teaching younger students.

### “Being and feeling different from peers”

“It bothers me sometimes, being an exception. I am insecure because you can see that we are very tall or very thin, and people will notice the dent in my chest, and ask me “what’s wrong with you?”; It’s not always nice, having to tell them you have Marfan’s, and I have to keep telling them over and over.” A4

#### Appearance

**Fig. 1:**
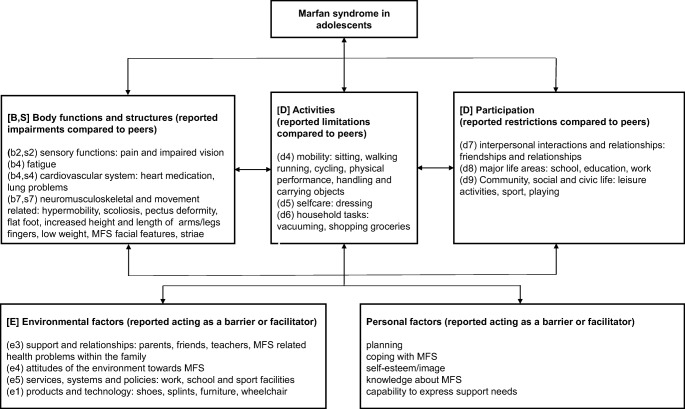
An adolescent Marfan syndrome-specific International Classification of Functioning, Disability and Health for Children and Youth (ICF-CY) model derived from the data describing the adolescent perceived impact of Marfan syndrome on (physical) functioning, disability and its contextual factors. The ICF-CY uses an alphanumeric coding system. The letters are according to the ICF-CY: “B” for Body function, “S” for Body structures, “D” for Activities/Participation and “E” for Environmental factors and are followed by a numeric code that starts with the chapter number of one digit [6]

Adolescents described themselves as different from their peers due to their appearance, fatigue and pain problems. They described their appearance as different (see Fig. 1).

#### Disability

Adolescents perceived limitations in activities compared to peers. They reported limitations in mobility, household tasks and physical/sport activities compared to peers (see Fig. 1). These limitations were variable among adolescents and depended on the severity of physical impairments, pain and fatigue.

#### Feeling different from peers

Adolescents indicated feeling different from peers because of their MFS appearance and disability. Bullying enhanced their negative feelings about themselves and their peer status. Some adolescents reported a negative self-image/esteem and as a consequence avoided social activities such as going to the swimming pool, beach, parties or sports activities.

### Contextual factors

Adolescents reported environmental and personal factors acting variably as a barrier or facilitator on the identified themes (see Table 3).

#### Coping with MFS

Adolescents reported positive and negative coping strategies to handle the impact of MFS on their (physical) functioning and disability. The reported positive coping styles, which seemed to improve their functioning, were pro-active coping, seeking social support, having a humorous and relaxed outlook on life, reappraising the disease and their disability in a positive light, acceptation and a healthy lifestyle. Most adolescents anticipated and planned their lives within their physical abilities to improve their participation in school, sports, leisure and work. Most adolescents sought social support from parents, friends and teachers, and most adolescents showed a humorous and relaxed outlook on their lives and did not worry much about medical issues. Some adolescents reassessed their negative associations about their MFS appearance and reported their tall stature as a benefit in sports like basketball and in getting into pubs at an early age. Adolescents reported better self-image and acceptation of their disability when they got older. Most adolescents were aware of the importance of a healthy lifestyle and incorporated physical activities in their weekly schedules.

Reported negative coping styles were avoidance and denial. Some adolescents avoided social activities with peers because they could not keep up or because of their low self-image. Furthermore, some adolescents did not want to know about MFS and future consequences.

#### Self-image/esteem

Adolescents reported that acceptance of their appearance helped them to feel accepted and less different from peers. Shame about appearance and fear of bullying acted as barriers for participation in social activities with peers.

#### Knowledge and future thoughts on MFS

Adolescents indicated that their knowledge of MFS was limited. In-depth questioning revealed knowledge about the development of physical impairments, (physical) functioning and disability and thoughts about a possible negative impact of MFS on school, sports, work, friendships and relationships and of their future offspring. Adolescents were mostly informed by their family, some of whom were diagnosed with MFS, and medical professionals. The Internet, other adolescents with MFS or the MFS patient support group were sometimes consulted. Most adolescents had limiting thoughts about their future, related to MFS or in general. Those with family members with complicated health problems due to MFS reported more awareness.

#### Ability to express support needs

In general, adolescents were satisfied with the current MFS (medical) support. They had difficulties expressing their support needs because they did not exactly know what type of support is available, but ultimately were able to state several support needs (see Table 4).

**Table 4:** Adolescents’ reported support needs

Adolescents’ reported support needs. They asked for advise on:	
(1) Improvement of fatigue, pain and physical impairments	
(2) Improvement of physical and sports activities	
(3) Pro-active planning of school and other activities	
(4) Safe and fitting sports activities	
(5) Fitting temporarily site-jobs	
(6) Fitting higher education	
(7) Future work possibilities	
(8) Organizing feasible social activities with peers	
(9) Support programmes on self-esteem and body image	
(10) A healthy diet to gain weight	
(11) Writing material/typing	
(12) Furniture, clothing, shoes and splints	
(13) Easy access to websites and educational programmes about MFS for themselves and for their families, friends and teachers	
(14) Contact groups with other adolescents with MFS	

#### Support from parents, friends and school

All adolescents reported that support from family, friends, teachers, medical staff and related health professionals facilitated participation in school, sports, leisure, chores, work and friendships/relationships. Families took into account the MFS-related limitations and restrictions in scheduling and planning leisure time and holidays, and stimulated adolescents to participate in activities with peers. Adolescents also perceived support from friends, such as texting during medical check-ups or invitations for (physically) achievable activities. Some teachers and employers provided excellent support and facilitated customised school programmes and work activities. On the other hand, some adolescents perceived little or no support from friends and teachers, which acted as a barrier to participate in school, sports and peer activities.

#### Peer group acceptance

Adolescents reported having best friends in school and their environment which made them feel accepted. Nevertheless, most adolescents reported the feeling of standing out from their peers because they could not always keep up with their peer group. They also reported bullying about their MFS appearance and/or limitations and restrictions in their (physical) functioning. Adolescents reportedly stay quiet about their disease, in order to be accepted by their peers, although some would inform classmates about their disease, (physical) functioning and disability.

## Discussion

This qualitative study showed that adolescents with MFS perceived limitations and restrictions in (physical) functioning. Indicated specific themes were (1) difficulties in keeping up with peers and (2) being and feeling different from peers due to their appearance and disability. Furthermore, an adolescent MFS-specific ICF-CY model of (physical) functioning and disability with its contextual factors acting as a barrier of facilitator derived from the data and adolescent support needs was categorized.

### Themes

The theme “difficulties in keeping up with peers” we identified is supported by earlier studies of adolescents and young adults with MFS who reported significant burden on schooling and job opportunities [33] and low work participation [34]. Also in adolescents and children with related congenital heart and connective tissue diseases, the negative impact of disease on school attendance and extracurricular activities [9], play, leisure [4] and school participation [15] were reported. Our study data add restrictions for higher education, work, sports, leisure and friendships/relationships in adolescents with MFS. Therefore, we recommend to address these topics during counselling.

The other theme “being and feeling different from peers” we identified is in line with studies in adolescents with MFS that reported limitations in physical activities [7, 27, 30] and self-image [27]. One systemic review concluded a significant impact of MFS on psychosocial aspects in adults: decreased quality of life; challenges in education, work and family life, depression and anxiety [35]; a correlation was found between perceived discrimination or socially devaluation because of having MFS and depressive symptoms, low self-esteem, physical impairments and perceived workplace discrimination [25]. Since being and feeling different from peers hinders (physical) functioning, we advise to discuss this subject with each adolescent with MFS.

### Adolescent MFS-specific ICF-CY model and contextual factors

An adolescent MFS-specific ICF-CY model derived from the data describing the adolescent perceived impact of MFS on (physical) functioning, disability and its contextual factors. Support of parents, friends and teachers was reported as a facilitator to participation with peers by our participants. A systematic review also concluded that support and positive relationships with peers contributed significantly to the participation of adolescents with congenital or acquired disorders [11]. Moreover, support of family helped adolescents with congenital heart disease to adopt a positive perception toward participation in activities [20]. Therefore, parents, friends and family should be made aware of the positive effect of support on (physical) functioning.

Our study shows that the contextual factor “knowledge about MFS” was limited in our participants, which could explain the limiting thoughts about their future life with MFS and the difficulties they had in expressing their support needs. A study on children and adolescents with chronic heart disease [5, 17], asthma and epilepsy [10] also showed knowledge gaps. Education about appearance, functioning and disability of MFS might help as a positive coping strategy and stimulate discussions about the adolescent (physical) functioning, which in turn may improve their ability to formulate support needs. This is supported by a study on adults with MFS that reported education on MFS as a positive coping strategy [25].

### Functioning versus quality of life and life satisfaction

Although quality of life and life satisfaction are different constructs, they might relate to functioning and disability. Children and adolescents with MFS have, like adults with MFS [13, 24, 26], a high risk of impaired health-related quality of life [14]. One study reported an unimpaired quality of life in adolescents [22] despite the distinctive phenotype, but children with symptoms related to the systemic Ghent score had a reduced quality of life and sub-scale scores on emotional well-being, compared to unaffected patients with MFS. The relationship between functioning and disability, quality of life and life satisfaction is worth investigating [16].

### Study strength, limitations

The strength of our qualitative study is that it is the first to describe adolescents’ perspectives on the impact of MFS on (physical) functioning, disability, contextual factors and support needs, which is not frequently addressed in the literature. The trustworthiness, credibility, content saturation and verification of this study were guaranteed throughout the entire study period.

Our study has some limitations. First, adolescents were not sent a resume of their interview. Second, all participants had the Dutch nationality and were treated in the Amsterdam University Medical Centers, an expertise centre for paediatric Marfan and related collagen diseases. Nevertheless, we assume that the results apply to adolescents with MFS and provide insights into assessments in other countries to observe intercultural differences.

### Clinical implications and further research

Our qualitative study contributed to themes on (physical) functioning and disability important to adolescents with MFS and to a complete overview of the adolescent’s perceived impact of MFS on (physical) functioning and disability. These themes and the adolescent MFS-specific ICF-CY model may be helpful in communication with the adolescents, relatives and medical staff as well as related health professionals about keeping up with peers, perceived being and feeling different from peers, and their (physical) functioning and disability. The adolescent MFS-specific ICF-CY model will also help identify (physical) functioning and disability within the acting contextual factors (barrier or facilitator) in the individual adolescent with MFS. We recommend a needs assessment and individual counselling on (physical) functioning, disability, contextual factors and support needs for every adolescent with MFS. This could prevent current and future disability.

The data from this study will be used to compile a core set of surveys and physical measurements regarding (physical) functioning and disability. With the knowledge obtained from these assessments, we aim to get qualitative and quantitative determinants of (physical) functioning and disability as well as acting contextual factors. This will enable us to develop customised physical, psychosocial, educational and environmental support programmes to improve (physical) functioning, empower adolescents with MFS and improve MFS health care.

## Electronic supplementary material

ESM 1 (PDF 225 kb)

## Abbreviations

ICF-CY: International Classification of Functioning, Disability and Health for Children and Youth
MFS: Marfan syndrome
